# Exploring stimuli-responsive elastin-like polypeptide for biomedicine and beyond: potential application as programmable soft actuators

**DOI:** 10.3389/fbioe.2023.1284226

**Published:** 2023-10-30

**Authors:** Yeongjin Noh, Eunjoo Son, Chaenyung Cha

**Affiliations:** Center for Multidimensional Programmable Matter, Department of Materials Science and Engineering, Ulsan National Institute of Science and Technology (UNIST), Ulsan, Republic of Korea

**Keywords:** elastin-like polypeptide, biomedicine, stimuli-responsiveness, soft actuator, shape deformation

## Abstract

With the emergence of soft robotics, there is a growing need to develop actuator systems that are lightweight, mechanically compliant, stimuli-responsive, and readily programmable for precise and intelligent operation. Therefore, “smart” polymeric materials that can precisely change their physicomechanical properties in response to various external stimuli (e.g., pH, temperature, electromagnetic force) are increasingly investigated. Many different types of polymers demonstrating stimuli-responsiveness and shape memory effect have been developed over the years, but their focus has been mostly placed on controlling their mechanical properties. In order to impart complexity in actuation systems, there is a concerted effort to implement additional desired functionalities. For this purpose, elastin-like polypeptide (ELP), a class of genetically-engineered thermoresponsive polypeptides that have been mostly utilized for biomedical applications, is being increasingly investigated for stimuli-responsive actuation. Herein, unique characteristics and biomedical applications of ELP, and recent progress on utilizing ELP for programmable actuation are introduced.

## 1 Introduction

Soft robotics as a scientific field has grown tremendously over the last two decades. Compared to the traditional robotics that involves the design and fabrication of controllable electromechanical parts with hard materials such as metals and hard plastics, soft robotics rely more on flexible and mechanically compliant materials that allow much greater complexity in movement and device fabrication Kim et al. (2013). The importance of soft robotics is tied to the growing need for the robots to adapt and function under dynamic 3D environment (e.g., temperature, humidity, electromagnetic field, and topography) and safely interact with human and other sensitive objects. Coyle et al. (2018) Therefore, flexible polymeric materials whose mechanical properties and stimuli-responsive transformation can be tuned in a wide range are generally selected Shen et al. (2020). In addition, soft robotics are ideal for creating miniaturized devices usually intended for wearable biomedical devices Cianchetti et al. (2018).

More recently, stimuli-responsive and compliant materials are actively investigated for biomedical applications, such as drug delivery, biosensor, and tissue engineering, as they can impart complex control over their functionalities, which are not generally possible for conventional materials. For example, drug release from soft materials, such as hydrogels and nanovesicles as drug delivery systems, is governed by the laws of diffusion. Even though the release rates can be controlled to a certain degree by controlling the mechanics of the materials, the release is continuous and not amenable for “switching on and off” under desired conditions. The stimuli-responsive polymers that undergo reversible phase transition between swollen and collapsed states under aqueous environment in response to changes in external stimuli, such as pH, temperature and ionic strength, have been widely adopted to develop the materials that allow more precisely block or facilitate the drug release at specific conditions Wei et al. (2017). The same stimuli-responsive materials can be used to detect biomarkers, as the binding of the biomarker elicit the change in the degree of stimuli-responsiveness in a scalable manner Shu et al. (2020).

A crucial factor for developing soft robotic systems is the proper actuation mechanism, which is also directly related to the type of material. El-Atab et al. (2020); Ilami et al. (2021); Li et al. (2022) The actuation of traditional robotics is mostly accomplished by integrating electromechanical and/or hydraulic devices to induce movement, so the type of material is generally not a primary concern. On the other hand, the material itself must act as the actuator for soft robotics, for the sake of developing compact and compliant devices that are able to change their physicomechanical properties in response to external stimuli without the need for separate actuators. For this reason, much of the research effort has been geared towards developing stimuli-responsive polymers, most notably electroactive polymers (EAP) and shape memory polymers (SMP) which undergo physical transformation upon change in temperature and electric field, as “soft actuators” Bar-Cohen, (2005); Lendlein, (2018); Xia et al. (2021); Maksimkin et al. (2022). For biomedical applications, thermoresponsive polymers, such as poly (N-isopropylacrylamide) (PNIPAm) and poloxamers [i.e., block copolymers consisting of poly (ethylene glycol) and poly (propylene glycol) blocks], have been especially popular, as their transition temperatures nearing temperature could be effectively utilized to generate implantable biomedical devices Roy et al. (2013); Bordat et al. (2019).

## 2 Overview of stimuli-responsive polymers as soft actuators

For the actuator to undergo highly specific changes, the material must be designed so that there is a precise correlation between the amount of stimulation and the degree of shape deformation. In addition, this correlation must be completely reversible for repeated operations. This means it should be possible to “program” their stimuli-responsive physical change, while maintaining their structural integrity and processability.

### 2.1 Electroactive polymers

**FIGURE 1:**
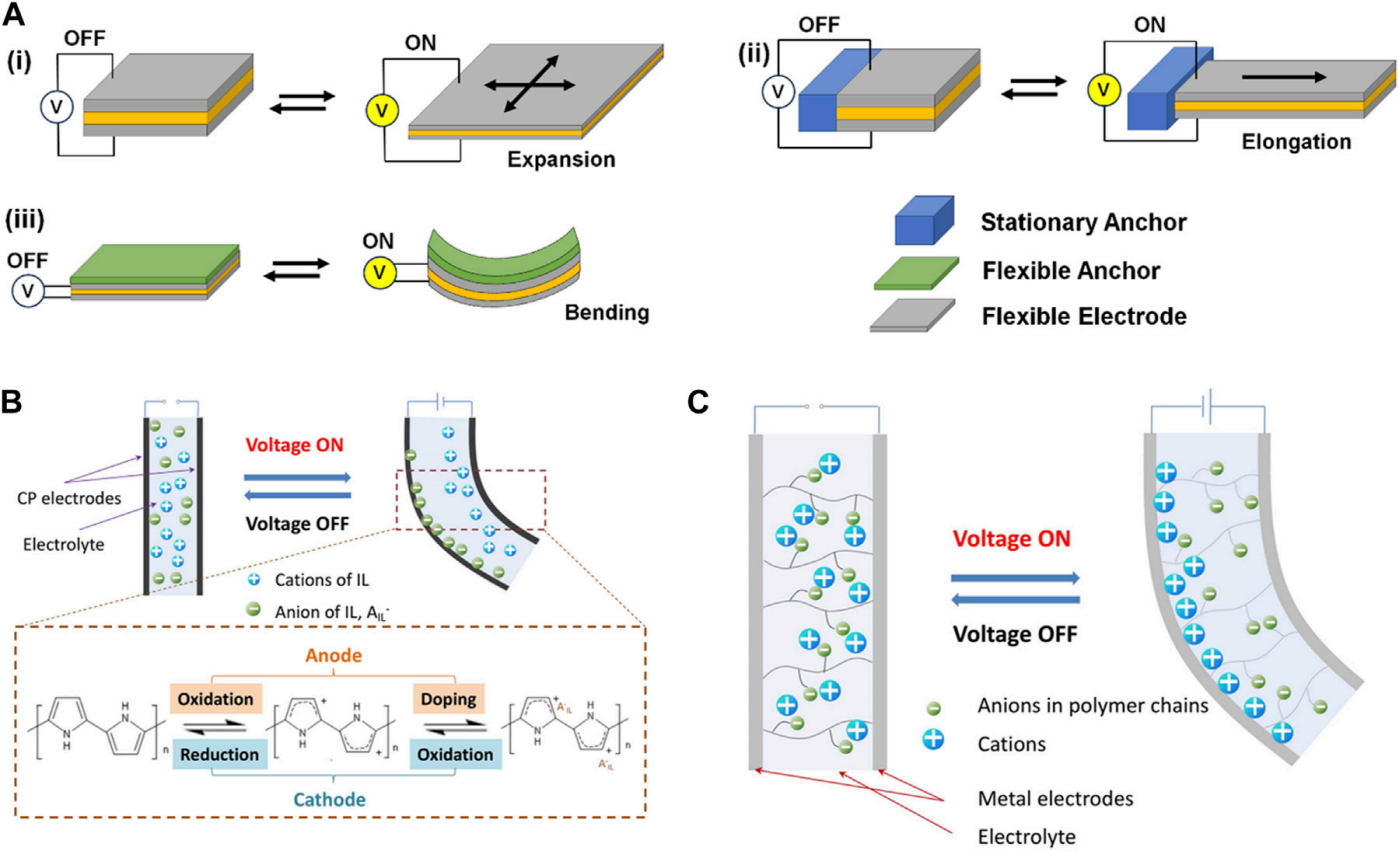
Schematic illustrations of actuators based on electroactive polymers undergoing shape deformation upon external electric field: **(A)** dielectric elastomer actuators, **(B)** conducting polymer actuator, and **(C)** ionic polymer-metal composite actuator. **(B,C)** are reproduced with permission from Dong et al. (2021), under the Creative Commons Attribution (CC-BY-4.0) license.

EAPs are defined broadly as the materials that can change their physical dimensions in response to externally applied electrical field Bar-Cohen and Anderson, (2019); Maksimkin et al. (2022). EAPs can be categorized into dielectric (“dry”) and ionic (“wet”) types. The electric field applied to a thin dielectric material creates molecular rearrangement and charge polarization. The charge accumulation on opposite ends generates repulsive force, causing the material to deform (Figure 1A). For example, poly (vinylidene fluoride) (PVDF) is a well-known dielectric polymer having piezoelectric and ferroelectric properties. Due to these electromechanical properties, PVDF has been one of the most investigated EAPs for actuators. Dielectric elastomers such as polysiloxane are also widely used because of their flexibility and mechanical compliance allowing larger strain Novelli et al. (2023). For the actuator to deform freely in all directions, the electrode also must be flexible enough to undergo similar shape deformation. In addition, the actuators anchored to stationary or flexible object can undergo different degrees of deformation.

Ionic EAPs rely on the mobility of ions embedded in the polymeric material under the electric field Chang et al. (2018); Neuhaus et al. (2020); Dong et al. (2021). For example, a tri-layer actuator consisting of two conductive polymer layers (e.g., polypyrrole) separated by an electrolyte layer undergo oxidation at the anode and reduction at the cathode (Figure 1B). The oxidized conductive polymer attracts counteranions from the electrolyte layer, causing the layer to expand and the entire structure to deform. Ionic polymers have also been explored as actuators, in the form of ionic polymer-metal composites (IPMC) and ionic polymer gels, which also involve asymmetric accumulation of ions (Figure 1C). Ionic EAPs can be operated under low voltage and produce large strain, but the response time is relatively slow, as they involve the diffusion of ions in liquid media. Dielectric EAPs, on the other hand, require relatively high electric field for polarization and the resulting strain is low, but their response time is faster. Regardless of type of materials, EAPs must accompany electrodes to supply electric field, so they are not generally feasible for creating miniaturized devices.

### 2.2 Thermoresponsive polymers

**FIGURE 2:**
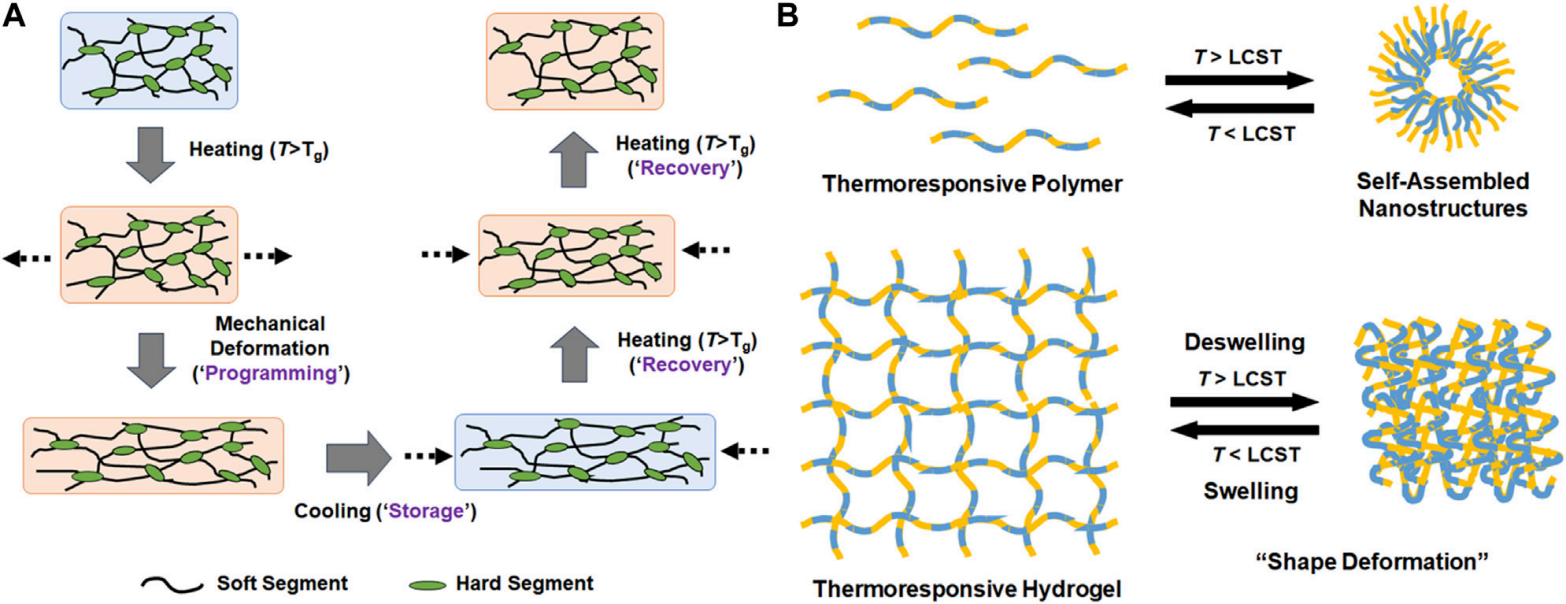
Schematic illustrations of the mechanisms of **(A)** shape memory polymers and **(B)** thermoresponsive self-assembled nanostructures and reversible swelling and deswelling of hydrogels.

In addition to electrical field, temperature has been another potent stimulus for inducing physical changes of soft actuators. Tokudome et al. (2016); Agarwal et al. (2019) Temperature change can be induced externally by using electrical heater/cooler systems. Alternatively, natural difference in temperature can be used to drive the actuation, such as bodily temperature for wearable/implantable device. Therefore, thermoresponsive materials that undergo physical change in response to temperature change have also been extensively studied as soft actuators. SMPs are the most widely explored class of programmable materials Ratna and Karger-Kocsis, (2008); Scalet, (2020); Xia et al. (2021). SMPs do not refer to a specific type of polymers, rather the polymers that can be deformed, or “programmed,” to a certain shape at an elevated temperature are induced to revert back to its original shape in response to a stimulus, usually temperature (Figure 2A). For a polymer to demonstrate the shape memory effect, it must have both the “soft” segment that can undergo stress-induced deformation usually above the glass transition temperature (*T*_g_), and the “hard” segment that holds its original position to which the deformed polymer can revert. Therefore, thermoplastic polymers engineered to form physical or chemical crosslinking are generally explored as SMPs. This actuation mechanism of SMP is entropically driven to the original disordered state. The most notable class of SMPs is polyurethane (PU) Huang et al. (2010); Menon et al. (2019). PU is generally synthesized by step-growth polymerization between diisocyanate and diol. The urethane bonds are capable of hydrogen bonding, which provides physical crosslinking. Most diisocyanate monomers, such as 4,4′-methylenedifenil diisocyanate, isophorone diisocyanate, and toluene diisocyanate, are rigid and serve as “hard segment” in PU to provide mechanical strength, while diols (or polyols) serve as “soft segment.” By tailoring their combination, thermoplastic and elastomeric PUs having a wide range of physicomechanical properties can be prepared. However, the shape change is not naturally reversible, and it must be mechanically reprogrammed at the elevated temperature for repeated performance.

Thermoresponsive polymers that can undergo reversible phase transition at a particular temperature, called lower critical solution temperature (LCST), have received significant research interest (Figure 2B) Zhang et al. (2019). These polymers remain soluble in aqueous media at lower temperature surrounded by water molecules via hydrogen bonding, but strong intermolecular interaction between polymer chain usually via hydrophobic interaction dominates at higher temperature leading to chain collapse, diminished solubility, and the formation of self-assembled nanostructures above a certain concentration. When these polymers are integrated into hydrated structures such as hydrogels, this thermoresponsive phase transition can induce a shape deformation. One of the first and most widely investigated example is PNIPAm, whose thermoresponsive phase transition is driven by hydrophobic isopropyl pendant groups Liu et al. (2022). The LCST of PNIPAm is near bodily temperature (at 32°C), which makes it ideal for biomedical applications. Many structural analogs of PNIPAm, which contains functional groups capable of similar attractive physical interactions, have been developed over the years having a wide range of LCST’s Roy et al. (2013). Block copolymers consisting of hydrophilic and hydrophobic blocks are another class of thermoresponsive polymers that can undergo phase transition via hydrophobic interaction. For example, poloxamers are triblock copolymers consisting of one hydrophobic poly (propylene glycol) midblock and two hydrophilic poly (ethylene glycol) endblocks that also demonstrate reversible phase transition whose LCST’s can be tuned widely Zarrintaj et al. (2018); Zarrintaj et al. (2020). Unlike PNIPAm, block copolymers can form nanostructures or hydrogels by themselves via self-assembly above LCST at different concentrations.

The most important attribute of thermoresponsive polymers is the reversibility of actuation, which is a clear advantage over SMPs that need repeated thermomechanical reprogramming. In addition, because of this innate reversible thermoresponsiveness, these polymers can be easily hybridized with other polymers and nanomaterials to impart the thermoresponsive actuation capability. However, because of the low mechanical strength, it is difficult to fabricate actuators by themselves, and as a consequence, they generally need to be integrated with the existing platform for mechanical reinforcement. Furthermore, the actuation mechanism for the thermoresponsive polymers is mostly based on hydrophobic interaction and hydrogen bonding which are mostly realized under aqueous environment. Therefore, thermoresponsive polymers are ideally suited for materials used for biomedical applications, such as hydrogels, nanofibers, and nanovesicles Wei et al. (2017); Shu et al. (2020).

With the continued maturation of the field of bioengineering, there is a growing trend to develop and adopt new materials having unprecedented functionality. In this regard, materials that can function as soft actuators are extensively investigated to impart biomaterials with controllable properties. In addition to known polymers such as PNIPAm, there is a new class of biopolymers, called elastin-like polypeptide (ELP), which are steadily emerging as a biocompatible, stimuli-responsive material system that possesses similar thermoresponsive properties, while allowing more sophisticated control of their properties via genetic engineering. In the following, the principles of genetic engineering technology to produce ELP and its basic properties are introduced. In addition, the notable examples of implementing ELP as a stimuli-responsive soft actuators for biomedical application are provided with a historical perspective.

### 2.3 pH and light-responsive polymers

As thermoresponsive polymers relied on the hydrophobic interaction at elevated temperature, there are polymers that can undergo phase transition in response to the changes in pH. For example, polyanions, the polymers containing numerous acidic functional groups such as carboxylic acid and sulfonic acid, can form hydrogen bonds at lower pH leading to chain collapse, while the deprotonation at higher pH leads to ionization and the loss of hydrogen bonding. Polycations, on the other hand, possess amino groups that acquire positive charge upon protonation at lower pH. In addition, these polymers can acquire charge at different pH’s, making them ionic EAP’s that can be actuated via external electrical potential. Olvera Bernal et al. develop a nanofiber-based soft actuator consisting of chitosan, a natural cationic polysaccharide, and poly (vinyl alcohol). Olvera Bernal et al. (2023) The nanofibers developed a net positive charge at lower pH due to the protonation of chitosan, thereby undergoing shape deformation in response to external electrical potential. Zhang et al. created a hybrid hydrogel consisting of cationic poly [2-(N,N′-dimethyl amino) ethyl methacrylate] (PDMAEMA) and anionic poly (acrylic acid) (PAAc) Zhang et al. (2020a). The hydrogels exhibited a wide range of pH-responsive swelling/deswelling based on the concentrations of PDMAEMA and PAAc.

There are photoactive polymers that can change their conformations in response to light at a specific range of wavelength. The most notable example is the polymer containing azobenzene moieties, which undergoes reversible trans-cis transition via UV light, called photoisomerization Mahimwalla et al. (2012); Chen et al. (2020). Compared to other stimuli, light has the advantage of more precise control of actuation by using a focused laser with a narrow wavelength. Xu et al. developed an azobenzene-containing liquid crystalline elastomer that can store the mechanical energy generated by trans-cis photoisomerization of azobenzene Lu et al. (2017). The release of mechanical energy by UV irradiation caused a large shape deformation.

## 3 Elastin-like polypeptide (ELP) as thermoresponsive biopolymer

It is truly fascinating to know that soft actuators based on EAP and thermoresponsive polymers are largely inspired based on human physiology. The movement of muscle tissue, contraction and relaxation of myofibers, is governed by the electrical signals relayed via neural network connected by neuromuscular junction, which is essentially the basis for EAPs Hannaford et al. (2001); Cui et al. (2020). A lesser-known fact about thermoresponsive polymer is that several types of bodily tissues also make use of a naturally thermoresponsive, fibrous protein, called elastin, to impart mechanical compliance and elasticity.

### 3.1 ELP: overview

**FIGURE 3:**
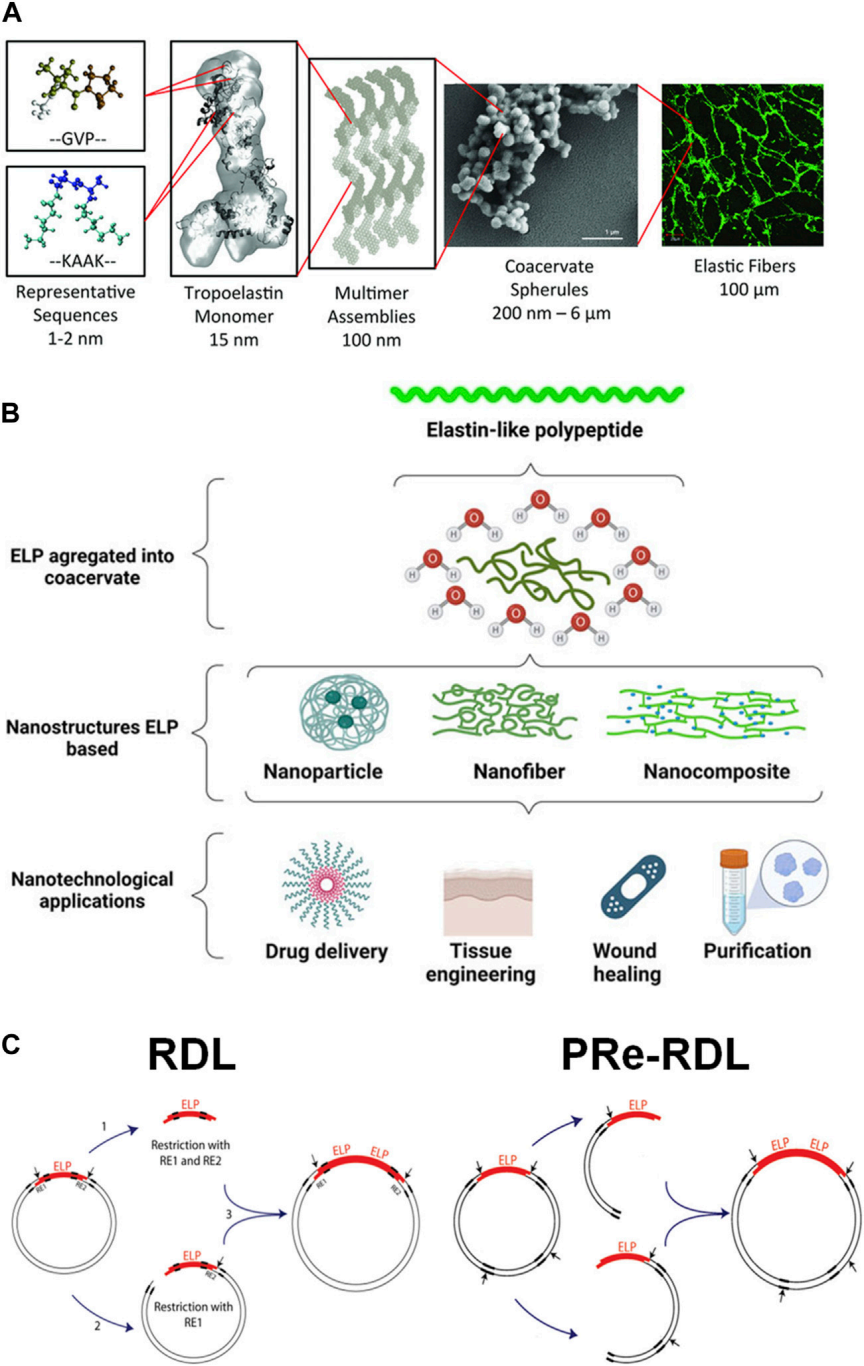
**(A)** The hierarchical assembly of tropoelastin molecules into crosslinked elastin fibers. Reproduced with permission from Tarakanova et al. (2019) under the Creative Commons Attribution (CC BY-NC 4.0) License. **(B)** The self-assembly of elastin-like polypeptide (ELP) to form various nanostructures for biomedical applications. Reproduced with permission from Lima et al. (2022) under Creative Commons Attribution (CC BY) License. **(C)** The construction of the plasmid DNA containing ELP gene by the conventional recursive directional ligation (RDL) or recursive directional ligation by plasmid reconstruction (PRe-RDL). Reproduced with permission from McDaniel et al. (2010). Copyright ^©^ 2010 American Chemical Society.

Elastin is found in many soft connective tissues, such as skin, blood vessel, tendon and ligament, as well as those in larger internal organs such as lung and heart Ozsvar et al. (2021). The common feature among these different tissues is the requirement for mechanical loading and compliance. Elastin is a crosslinked, fibrous network of large polypeptides (around 50–70 kDa) called tropoelastin (TE) (Figure 3A) Almine et al. (2010); Tarakanova et al. (2019); Rodriguez-Cabello et al. (2021). TE has a very characteristic amino acid sequence, consisting of repeats of non-polar, hydrophobic pentapeptide units, a Val-Pro-Gly-X-Gly motif with X being any amino acid residue besides proline. Abundance of proline makes TE demonstrate helical secondary structure. The unique aspect of TE is the abundance of valine containing isopropyl group, which is essentially a structural analog of PNIPAm. Therefore, much like PNIPAm, tropoelastin similarly demonstrates thermoresponsive chain collapse at elevated physiological temperature. These factors allow the tissue containing elastin fibers to undergo substantial mechanical deformation in response to external force without structural damage Ozsvar et al. (2021).

Because of this thermoresponsive properties as well as biocompatibility, there have been significant research efforts to utilize elastin for biomedical applications in addition to basic biochemical research (Figure 3B) Lima et al. (2022). However, it is technically challenging to extract elastin in its native form from biological tissue without structural damage in large quantities. Therefore, the production of TE via recombinant DNA technology has been viewed as a viable alternative. Sequencing of the mRNA and construction of different variations of complementary DNA (cDNA) of TE have been developed over the years Indik et al. (1987); Fazio et al. (1988); Martin et al. (1995). Recombinant tropoelastin (rTE) that retain their characteristic features (e.g., thermoresponsiveness, elasticity, enzymatic crosslinking, and cellular and biomolecular adhesions) could be produced in large quantities using the cDNA to implement recombinant technology Indik et al. (1990); Bedell-Hogan et al. (1993); Wu et al. (1999). More recently, rTE has been increasingly utilized for biomedical applications, including drug delivery and tissue engineering Wise et al. (2009). For example, Annabi et al. developed a mechanically-tough and biocompatible nanocomposite hydrogel consisting of graphene oxide embedded in radically crosslinked rTE hydrogel with methacrylate-functionalized rTD Annabi et al. (2016). The presence of GO improved not only the mechanical strength, but also the electrical conductivity, which provided optimal environment for cardiomyocyte functions.

Despite the recent success in the synthesis and application or rTE, there are several limitations that prevent more efficient production and application of rTE. The biosynthesis of rTE via recombinant technology is often technically challenging, as rTE often becomes degraded by the bacterial host or bacterial membrane, often requiring fusion with other protein using lysogenic host Indik et al. (1990). Since the rTE is mostly based on the native TE genes, it is not amenable to control the molecular weight and introduce other functional groups to modulate the physicomechanical and bioactive properties of resulting materials. Alternatively, more recent research trend of recombinant technology for elastin has shifted its focus to developing polypeptides containing the characteristic pentapeptide moieties, now universally termed elastin-like polypeptide (ELP), rather than its full sequence. The first reported ELP was developed by solid-state peptide synthesis, which is not amenable to generating high molecular weight species with accuracy. Martino et al. (2000) Like rTE, the recombinant technology has become the standard method of producing ELP. Unlike rTE, on the other hand, the ELP expression can be accomplished using a variety of plasmids and transfection hosts, and it does not suffer from premature degradation and remain stable during the purification process Meyer and Chilkoti, (2002).

The advancement in the recombinant technology for ELP was remarkably boosted by a key experimental techniques, called recursive directional ligation (RDL), developed by Chilkoti and co-workers Meyer and Chilkoti, (2002). It is often challenging to ligate a large gene into a plasmid. Because of the repetitive nature of ELP sequence, it is more efficient to construct a plasmid by inserting multiple copies of oligomers by repeated digestion and ligation. In a typical RDL process, one set of the parent plasmid vector containing ELP oligomeric insert is fully digested to obtain the oligomer insert, while the other set is only digested on one side of the oligomer to become a linearized vector (Figure 3C) McDaniel et al. (2010). The insert is then ligated with the linearized vector to reconstruct the plasmid vector with the dimerized gene. This process can be easily repeated to build a larger vector. More recently, a modified version of this technique has been developed, called recursive directional ligation by plasmid reconstruction (PRe-RDL), which could overcome the limitations of RDL McDaniel et al. (2010). Since RDL involves the full digestion and purification of the insert, it is quite time-consuming. Also, the restriction site required for this process could limit the choice of codons. The inserts can also self-ligate, making it difficult to control the number of repeats. In Pre-RDL, instead of utilizing inserts, two sets of parent plasmids are only digested on one side, and thus, without generating the inserts, ligated together. Therefore, the gene length (the number of repeats) can be precise controlled. Also, it can be used to efficiently introduce other genes expressing desired functional peptides. Pre-RDL has been proven highly useful for synthesizing a plasmid having a large, repetitive oligomeric gene McDaniel et al. (2010).

Another advantage of recombinant technology for ELP production is the simple purification process. Because of the thermoresponsiveness, ELP undergoes aggregation at higher temperature above LCST. Therefore, after cell lysis to dissolve the expressed ELP in the aqueous buffer, the temperature is increased above LCST to induce the ELP aggregation which is then collected via centrifugation. This process can be repeated to maximize the yield. This process, called inverse transition cycling, is by far more efficient and less arduous than a typical polyhistidine tagging and liquid chromatography. Meyer and Chilkoti, (1999).

### 3.2 ELP-based materials for biomedical applications

**FIGURE 4:**
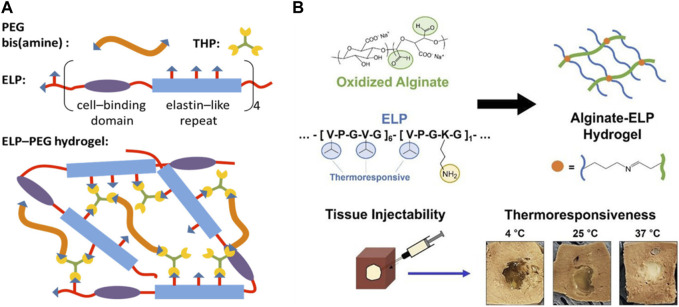
**(A)** Synthesis of poly(ethylene glycol) (PEG)-ELP hybrid hydrogel by crosslinking amine-functionalized PEG and ELP with tris (hydroxymethyl)phosphine (THP). ELP also contained cell-binding domain (RGD peptide) to utilize the hydrogel for tissue engineering applications. Reproduced with permission from Wang et al. (2014). Copyright ^©^ 2014 American Chemical Society. **(B)** Synthesis of tissue-injectable ELP-alginate hydrogel by Schiff base formation between lysine-rich ELP and aldehyde-presenting alginate. The hydrogel demonstrated hydrophobic phase transition at higher temperature, as evidenced by the decrease in size and increase in turbidity. Reproduced with permission from Lee et al. (2022b). Copyright ^©^ 2022 American Chemical Society.

The most important and distinguishable biological function of elastin is imparting elasticity and mechanical toughness to biological tissues. Naturally, ELP has been often adopted to generate a variety of biomaterials with improved mechanical and thermoresponsive properties for biomedical applications MacEwan and Chilkoti, (2010); Varanko et al. (2020). For example, Zhang et al. demonstrated that ELP hydrogel crosslinked by disulfide bonds of cysteine residues were highly extensible and showed little hysteresis against repeated mechanical stress Zhang et al. (2015). The biocompatibility of the hydrogel was confirmed by the high viability of mesenchymal stem cells (MSCs) and human umbilical vascular endothelial cells (HUVECs) cultured on the hydrogel, as well as the low immunogenic response after implantation into an animal model. ELP can also be hybridized with other polymers to tune the mechanical properties of resulting hydrogels. Wang et al. developed poly (ethylene glycol) (PEG)-ELP hybrid hydrogel by crosslinking amine-functionalized PEG and ELP with tris(hydroxymethyl)phosphine (Figure 4A) Wang et al. (2014). Mechanical stiffness could be controlled by the crosslinking density of PEG and ELP. In order to impart cell adhesion properties to hydrogel, RGD peptide was conjugated to ELP by simply the fusion of RGD gene alongside the ELP gene to the plasmid, which is a clear advantage of recombinant technology. By keeping the RGD-ELP constant, the cell adhesion and mechanical properties of the hydrogel could be independently controlled. Similarly, Lee et al. demonstrated hydrogel formation via Schiff base reaction between lysine-rich ELP and aldehyde-functionalized alginate (Figure 4B) Lee et al. (2022b). Since the reaction occurred spontaneously without additional chemicals, this type of thermoresponsive gelation is ideally suited as a tissue-injectable delivery system, in which biocompatible, thermoresponsive gelation is induced upon injection followed by sustained release of bioactive molecules. Zhu et al. created ELP-hyaluronic acid (HA) hydrogel by Schiff base formation between aldehyde-presenting HA and hydrazine-presenting ELP Zhu et al. (2017). Increasing HA concentration resulted in increased cartilage phenotypes and reduced fibrocartilage phenotypes of encapsulated chondrocytes.

Since ELP is synthesized by recombinant DNA technology, it is feasible to develop fusion protein with other polypeptides and proteins to acquire synergistic properties. López Barreiro et al. (2023) For example, silk fibroin is a natural protein mostly procured from silkworm increasingly used as scaffold material for biomedical applications. Similar to ELP, silk fibroin also contains characteristic peptide repeats (e.g., GAGAX, X: guest residue). Therefore, there have been research efforts to develop the fusion polypeptide, “silk-elastin-like polypeptide (SELP),” via recombinant technology in order to control the physicomechanical properties Huang et al. (2015); Gonzalez-Obeso et al. (2022). A similar strategy has been used to generate “collagen-elastin-like polypeptide” by incorporating the characteristic motif of collagen, glycine-proline-4-hydroxyproline Prhashanna et al. (2019).

**FIGURE 5:**
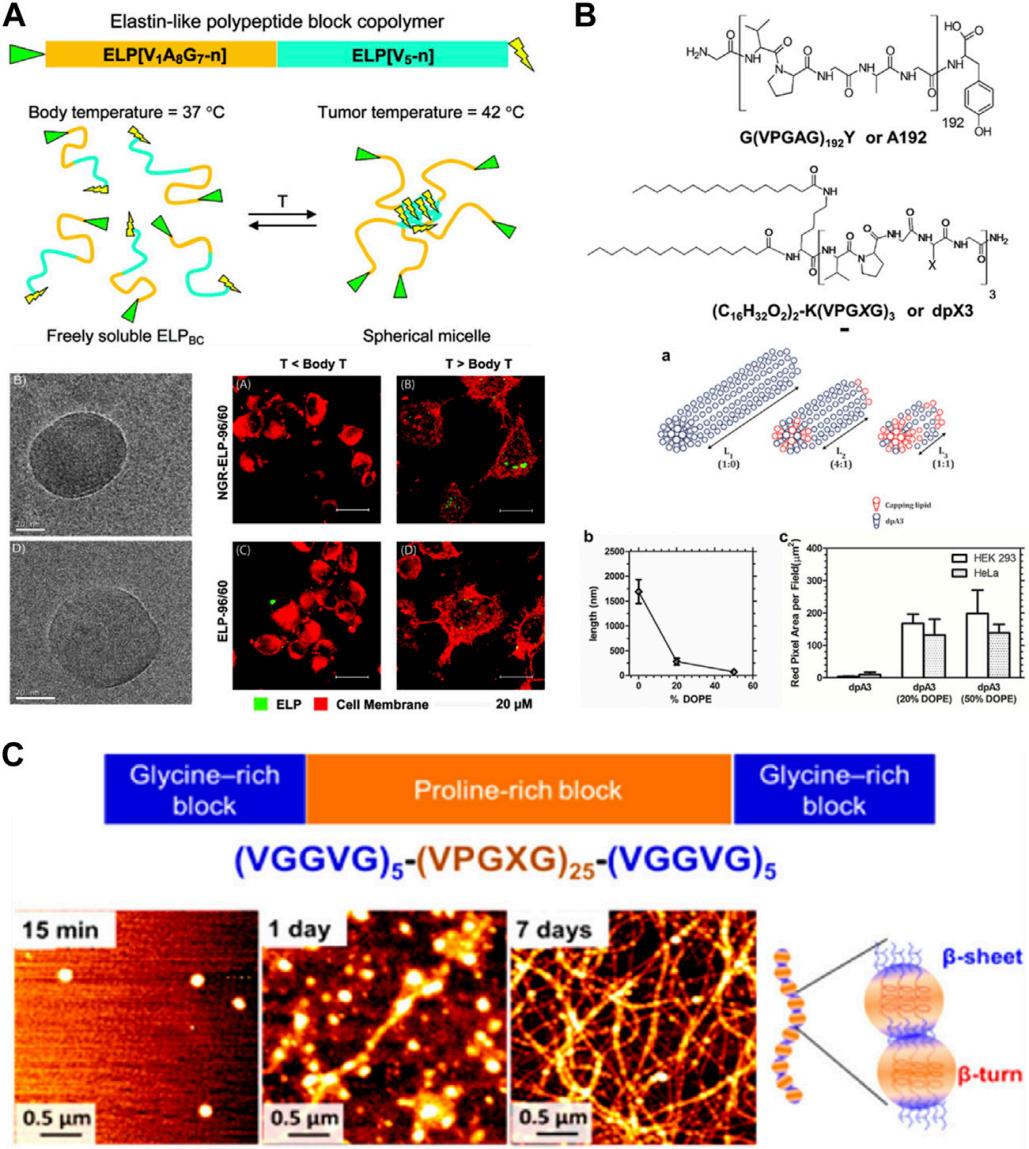
**(A)** ELP block copolymer consisting of alanine-rich hydrophilic and valine-rich hydrophobic blocks undergoing self-assembly to form micelles above body temperature. The micelles showed increased binding to tumor cells. Reproduced with permission from Dreher et al. (2008). Copyright ^©^ 2008 American Chemical Society. **(B)** ELP conjugated with a phospholipid, 1,2-dioleoyl-sn-glycero-3-phosphoethanolamine (DOPE), allowed the control of the size of self-assembled nanofibers. Reproduced with permission from Aluri et al. (2012). Copyright ^©^ 2012 American Chemical Society. **(C)** ABA-type triblock ELP copolymer consisting of proline (P)-rich midblock and glycine (G)-rich endblocks undergoing hierarchical assembly from micelles via beta-turn of P-blocks to nanofibers via beta-sheet formation of G-blocks. Reproduced with permission from Le et al. (2013). Copyright ^©^ 2013 American Chemical Society.

Like other biopolymers, ELP can be processed into nanofibers via electrospinning Lee et al. (2011); Putzu et al. (2016). ELP has a unique ability to form nanofibers, in addition to micelles and vesicles, by self-assembly of AB-type ELP block copolymers consisting of hydrophilic and hydrophobic blocks, taking advantage of its thermoresponsive properties and genetic engineering capability Saha et al. (2020). ELP block copolymers could be synthesized by modifying the guest residues of the pentapeptide units with either hydrophobic or hydrophilic amino acids. For example, alanine or glutamic acid having carboxylic acid is introduced as a guest residue to create hydrophilic blocks, while glycine, valine, or phenylalanine is introduced to generate hydrophobic blocks. Depending on the specific sequence and molecular weight, a wide range of nanostructures including nanofibers and micelles could be generated. For example, Dreher et al. developed ELP block copolymers consisting of alanine-rich hydrophilic and valine-rich hydrophobic blocks (Figure 5A) Dreher et al. (2008) This ELP block copolymer could self-assemble to form nanostructures above the body temperature. Interestingly, there were two distinct thermal transitions, which were attributed to the polymer-to-micelle and micelle-to-coacervate transitions. Furthermore, introducing tumor-targeting NGR peptide onto ELP helped facilitate the increased binding of ELP nanostructures to HT-1080 fibrosarcoma cells. McDaniel et al. induced self-assembled nanofiber formation of ELP copolymers by introducing the “assembly domain” to C-terminus of the alanine-rich hydrophilic domain. McDaniel et al. (2014) The assembly domain was a short hydrophobic domain, (XGG)_8_, where X was tyrosine, phenylalanine or tryptophan. The size of the nanofibers as well as their LCST’s could be effectively controlled by the type of X residue. These ELP-based nanofibers have been utilized as scaffolds for tissue engineering applications. Natsume et al. developed the self-assembled ELP nanofibers presenting cell-adhesive REDV peptide as a scaffold for vascular graft generation Natsume et al. (2023). This REDV-presenting nanofibers demonstrated reduced platelet adhesion, increased bioactivity of endothelial cells and smooth muscle cells.

Another advantage of thermoresponsive self-assembly of ELP nanofibers is the ability to precisely control their length and morphology, which is not generally feasible for electrospun nanofibers. These self-assembled nanofibers have been used to develop drug delivery vehicles and tissue engineering scaffolds. It is also possible to conjugate other polymers or lipids to tune nanofiber dimensions and morphologies. Aluri et al. demonstrated that the size of self-assembled nanofibers could be controlled by conjugating 1,2-dioleoyl-sn-glycero-3-phosphoethanolamine (DOPE), a phospholipid, to ELP (Figure 5B). Aluri et al. (2012) Increasing presence of DOPE provided stronger hydrophobic interaction, and as a result, led to shorter nanofibers. It has also been shown that even without hydrophilic blocks, nanofibers could be generated using ELP consisting of “double-hydrophobic” glycine (G)-rich and proline (P)-rich domains Le et al. (2013); Le et al. (2017). Le et al. demonstrated that the initial self-assembly by P-rich domain results in nanoparticle formation by beta-turn formation, while subsequent increase in b-sheet formation by G-rich domain connect the nanoparticles into a long nanofiber formation with beaded nanofiber morphology (Figure 5C).

**FIGURE 6:**
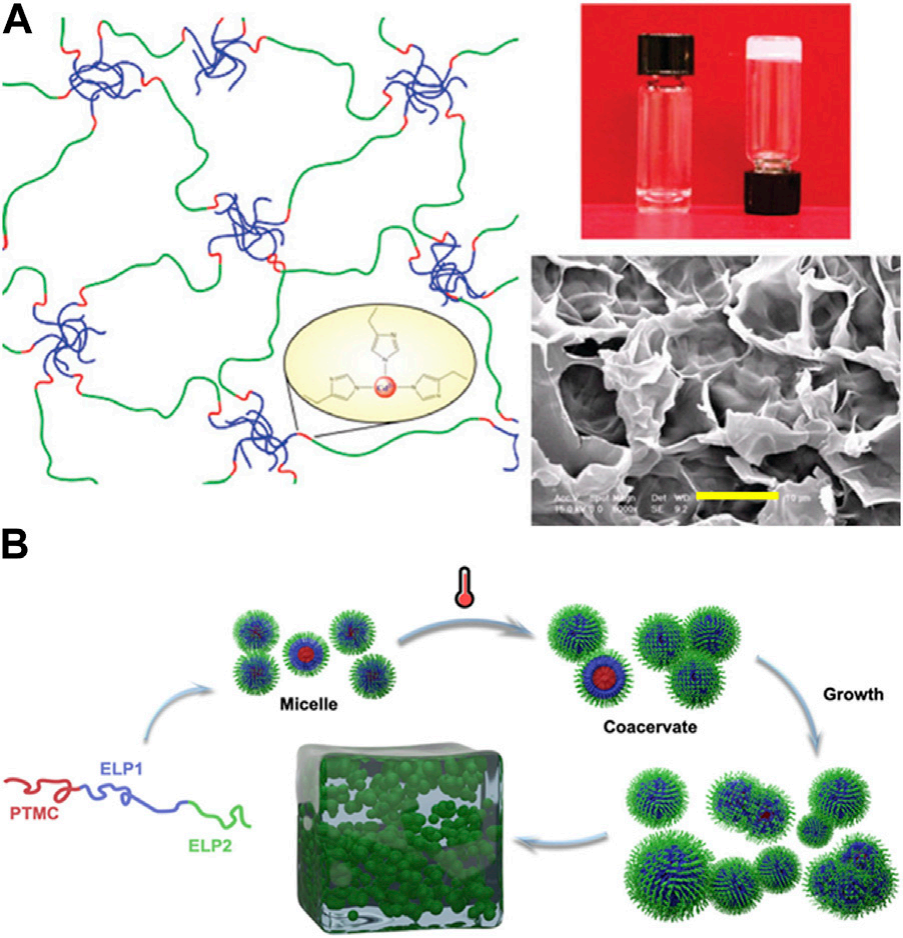
**(A)** Thermoresponsive hydrogel formation via self-assembly above a critical concentration of ABA-type triblock ELP consisting of valine (V)-rich hydrophobic endblocks and glutamic acid (E)-rich midblock (scale bar: 10 µm). Polyhistidine tag on the ELP allowed increased metal ion binding. Reproduced with permission from Lao et al. (2007). Copyright ^©^ 2007 American Chemical Society. **(B)** ABC-type ELP consisting of poly(trimethylene carbonate) (PTMC) block in addition to two ELP blocks, a methionine and valine-rich block and a isoleucine-rich block, resulted in the hierarchical assembly from micelles and larger coacervates to a macroscopic hydrogel. Reproduced with permission from Dai et al. (2021). Copyright ^©^ 2020 American Chemical Society.

ELP block copolymers above a certain critical concentration can form extended network structure via self-assembly to form hydrogels, similar to well-known poloxamers. For example, Lao et al. demonstrated thermoresponsive hydrogel formation of ABA-type triblock ELP consisting of valine (V)-rich hydrophobic endblocks and glutamic acid (E)-rich midblock, [(VPGVG)_2_VPGEG (VPGVG)_2_]_n_ above a critical concentration of 6% (w/v) (Figure 6A) Lao et al. (2007). The hydrogel formation was thermally reversible, remaining gel state above room temperature and reverting to sol state upon cooling (4°C). Furthermore, polyhistidine tag on ELP also enhanced the metal ion binding. Ghoorchian et al. demonstrated that the micelle made from AB-type diblock ELP containing zinc-binding motif Ghoorchian et al. (2015). The addition of zinc could induce hydrogel formation. In addition to AB-type diblock copolymers and ABA-type triblock copolymers, Dai et al. developed an ABC-type triblock copolymer consisting of a poly(trimethylene carbonate) (PTMC) block in addition to two ELP blocks having different degrees of hydrophobicity, a methionine and valine-rich block and a isoleucine-rich block (Figure 6B) Dai et al. (2021). Having additional degree of complexity in the ELP block copolymer allowed more intricate, hierarchical control of the morphology of resulting self-assembled structures, in which micelles first formed at low concentrations [0.1%–0.3% (w/v)] became larger coacervates at temperatures above the LCST. With further increase in concentration, the coacervates grew into micrometer-scale particles, which eventually undergo sol-gel transition to become macroscopic hydrogel [4% (w/v)].

### 3.3 Emerging role of ELP as thermoresponsive soft actuators: historical perspective and recent development

Naturally, being a polypeptide, most of the research on ELP has been focused on designing and implementing ELP for biomedical applications. The characteristic thermoresponsive properties are like those of widely explored PNIPAm, while the recombinant technology allows other peptide moieties to be integrated with ELP for added functionalities. This is a clear advantage over synthetic polymers like PNIPAm, which need to be hybridized with other functional materials. As PNIPAm over the recent years has been widely explored as thermoresponsive soft actuators, there has been a rising research trend in adopting ELP for this purpose as well.

Urry and co-workers were one of the first to introduce the possibility of the mechanical actuation of chemically-crosslinked ELP hydrogels Urry, (1988); Urry et al. (1988). The hydrophobicity-driven phase transition of ELP hydrogel, as identified by the changes in mechanics and dimensions, was demonstrated by modulating pH and temperature, which was termed “mechanochemical coupling.” While these earlier attempts at developing ELP hydrogels for soft actuation presented in these studies were fabricated by non-specific radical crosslinking via gamma irradiation, more systematic approaches were taken in subsequent studies to develop ELP hydrogel by taking advantage of presenting specific amino acid sequences. Trabbic-Carlson et al. developed ELP hydrogel demonstrating reversible, thermoresponsive swelling/deswelling by crosslinking lysine-rich ELP (i.e., lysine as the guest residue) with tris-succinimidyl aminotriacetate as the crosslinker Trabbic-Carlson et al. (2003). Controlling the concentration, molecular weight and lysine content of ELP allowed more systematic modulation of the mechanical properties, and subsequent thermoresponsive shape change, of the ELP hydrogels.

**FIGURE 7:**
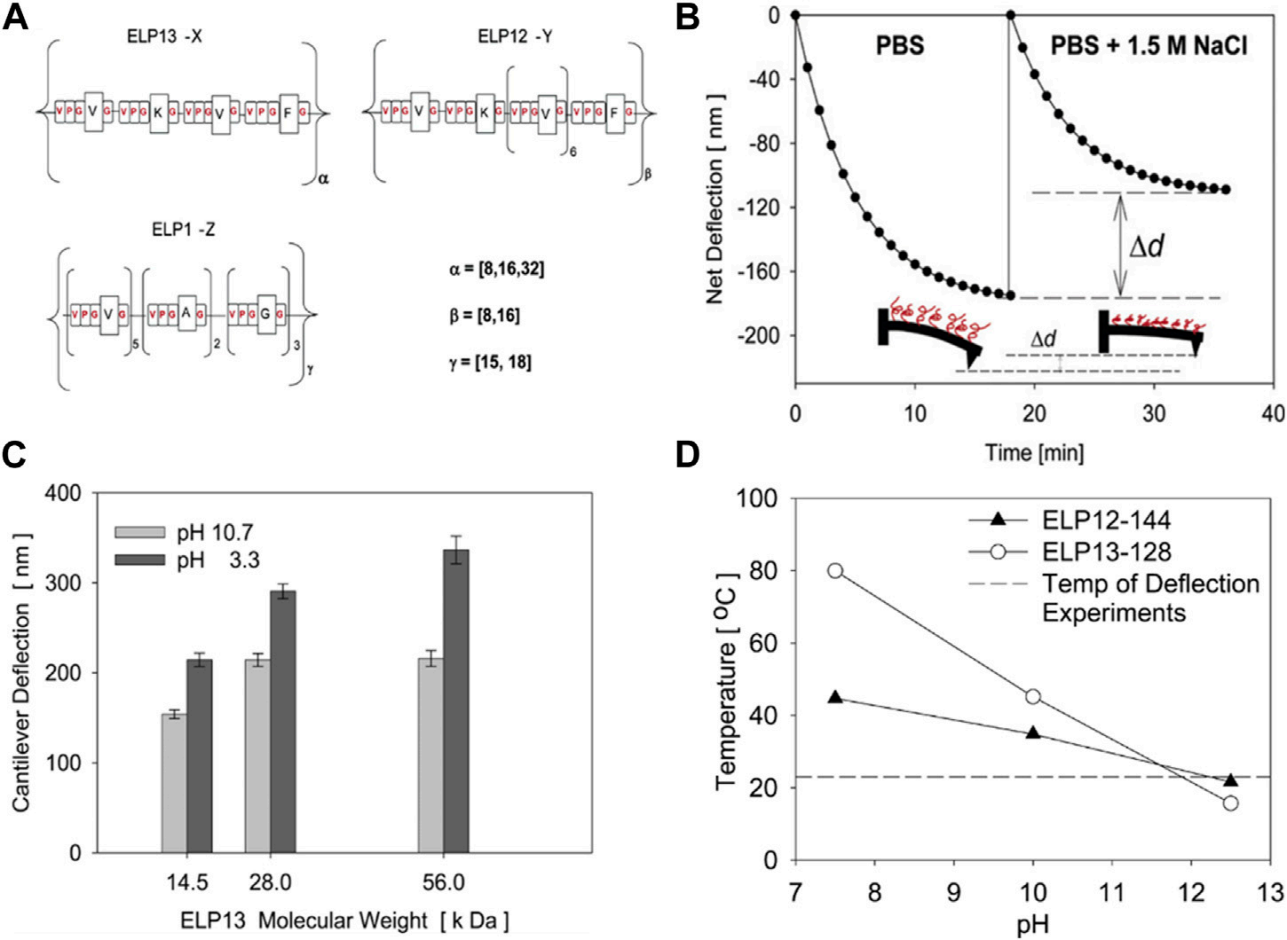
**(A)** ELP’s having different molecular weights and pentapeptide compositions were prepared and coated on the surface of the cantilever used for atomic force microscopy (AFM). The degree of deflection of the ELP-coated cantilever under different **(B)** ionic strengths and **(C)** molecular weights of ELP and pH. **(D)** The transition temperatures of ELP’s measured at different pH’s. Reproduced with permission from Valiaev et al. (2007). Copyright ^©^ 2007 American Chemical Society.

While the study by Trabbic-Carlson et al. focused more on the basic mechanical characterization of ELP hydrogels, Valiaev et al. demonstrated the practical potential of ELP to modulate the actuation of microcantilever (Figure 7) Valiaev et al. (2007). The silicon nitride microcantilever used for atomic force microscopy and quartz crystal microbalance was grafted with ELP with varying molecular weights and guest residue compositions. The change in the deflection of the microcantilever in response to various parameters, such as temperature, ionic strength, and pH, could be tuned by ELP’s with varying molecular weights and guest residues. The degree of cantilever deflection was increased at lower pH and higher molecular weight. Even though ELP was only used for surface functionalization, the tunable thermoresponsive nature of ELP alone could effectively control the degree of actuation of existing actuators.

One common method of producing a soft actuator is to attach two layers consisting of stimuli-responsive “active” layer and a non-responsive “passive” layer, so the actuator can bend in one direction upon stimulation in a controlled manner based on the difference in mechanics. Kamada et al. developed a bilayer actuator consisting of polyacrylamide (PAAm)-ELP hydrogel layer and non-crosslinked ELP hydrogel Kamada et al. (2023). The degree of actuation could be controlled by the concentration of ELP and the ionic strength of the medium.

**FIGURE 8:**
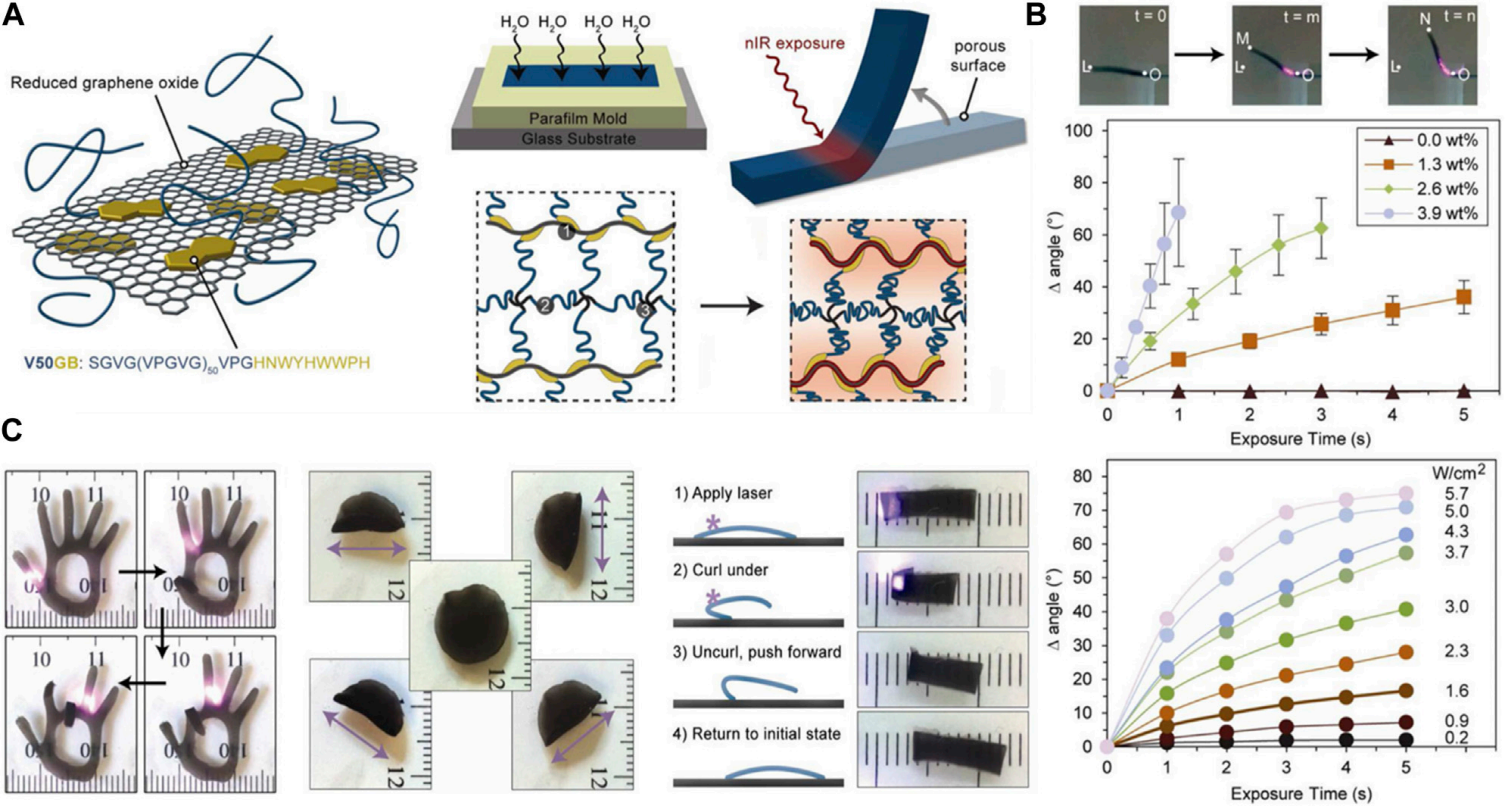
**(A)** Schematic illustration of the fabrication of near infrared (NIR)-responsive reduced graphene oxide (rGO)-ELP composite hydrogel actuator. **(B)** The degree of actuation (bending angle) controlled by the rGO concentration and NIR intensity. **(C)** The control of bending motions of various rGO-ELP soft actuators. Reproduced with permission from Wang et al. (2013). Copyright ^©^ 2013 American Chemical Society.

The ELP hydrogels that are structurally robust enough can also function as thermoresponsive soft actuators. Increasing the temperature above LCST to induce the shape deformation can be easily accomplished with a variety of heat sources. However, it is technically challenging to precisely control the thermoresponsive actuation in a localized manner using conventional heat sources. One innovative strategy to accomplish this task is by incorporating reduced graphene oxide (rGO) into the ELP hydrogel. rGO has been shown to display photothermal effect, in which thermal energy is generated upon photoexcitation using near IR (NIR) range. Wang et al. successfully demonstrated that the irradiation of NIR laser upon rGO-ELP nanocomposite hydrogel generated enough heat at the area of the irradiation, causing the hydrogel to display highly coordinated motions (Figure 8) Wang et al. (2013). The degree and speed of actuation could be enhanced by the concentration of ELP and the intensity of NIR laser. The rGO-ELP hydrogel was then used to fabricate soft robots capable of sophisticated light-induced locomotion by selective irradiation.

More recently, Chiang et al. presented a more expansive exploration of rGO-laden ELP hydrogel as a soft actuator mediated by the photothermal effect Chiang et al. (2021). They controlled the composition of tetrakis (hydroxymethyl) phosphonium chloride (TMPC)-crosslinked ELP hydrogels by introducing either graphene oxide (GO) or rGO. In addition, ELP was copolymerized with silk fibroin (SF) to develop hybrid hydrogel. Using this dual approach, more comprehensive control of the mechanical properties was possible. For instance, hybridizing with SF alone increased the rigidity of the hydrogel, but it also became more brittle. Incorporating GO or rGO into the ELP-SF hydrogel led to the significant increase in tensile strength and stretchability. They ultimately created a bilayer actuator consisting of one hydrogel layer containing GO and the other containing rGO. Because of the mechanical anisotropy between the layers, varying the ratio of GO to rGO could modulate bending motion of the bilayer actuator, which was further controlled by the intensity of NIR irradiation. Lo et al. demonstrated that the bioactivity of cardiomyocytes cultured on the rGO-laden ELP-SF hydrogel was well maintained, while being able to undergo the light-induced activation. Lo et al. (2017).

**FIGURE 9:**
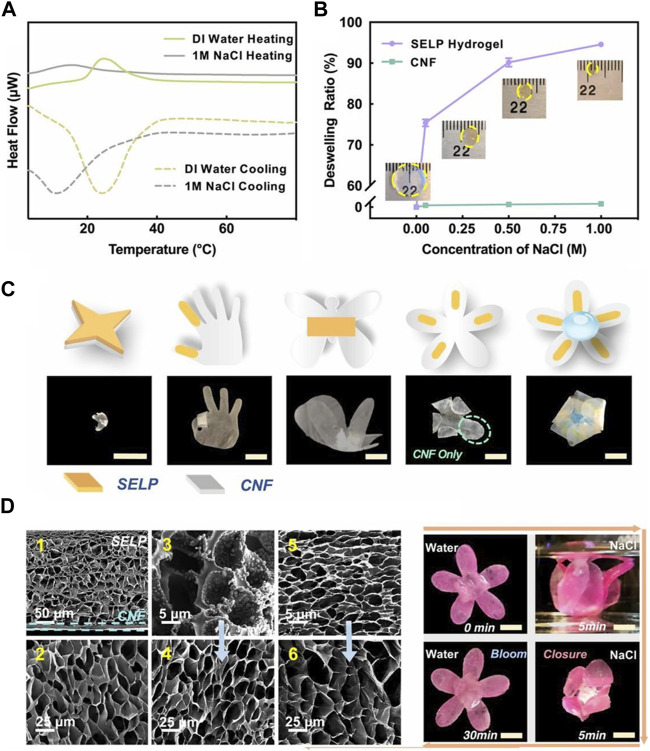
**(A)** Heat flux of silk fibroin-ELP (SELP) hydrogel measured in different ionic strength (deionized water vs. 1 M NaCl) by differential scanning calorimetry. **(B)** The deswelling ratios of SELP hydrogels measured under varying ionic strengths (up to 1 M NaCl). **(C)** The shape change of various soft bilayer actuators made of SELP hydrogel and cellulose nanofiber (CNF) membrane by 1 M NaCl. **(D)** Scanning electron microscopic images of (D1) SELP/CNF bilayer actuators and (D2) SELP hydrogels in swollen states at 4°C DI water, SELP hydrogels in contracted states at (D3) 1 M NaCl solutions at room temperature and (D5) 60°C DI water, respectively, and (D4 and D6) equilibrated back to 4°C DI water of (D3 and D5). Reproduced with permission from Wang et al. (2020). Copyright ^©^ 2020 National Academy of Sciences.

In addition to creating a hybridized network of silk fibroin and ELP by copolymerization, it is also possible to create a fusion protein of silk fibroin and ELP by recombinant DNA technology. While hybridization leads to randomized distribution of silk fibroin and ELP, it can be inferred that the silk fibroin unit directly attached to ELP would have more intimate effect on the thermoresponsive properties of ELP. Wang et al. developed a fusion protein consisting of the characteristic domain (GAGAGS) of silk fibroin and ELP (“SELP”), in which the silk domain provided greater hydrophobicity and mechanical strength (Figure 9) Wang et al. (2020). The thermoresponsive phase transition of SELP hydrogel was shown to be influenced by both temperature and ionic strength. For example, the LCST was lowered to 15°C from 25°C when 1 M NaCl was added to deionized water. Also, the thermoresponsive deswelling became more pronounced in the presence of 1 M NaCl at the same temperature. Using this behavior, the bilayer actuators consisting of the active SELP hydrogel layer and the passive cellulose nanofiber (CNF) layer were developed, which allowed highly sophisticated motions upon the changes in temperature and ionic strength. These findings suggested that providing a functional moiety to ELP via genetic engineering could modulate the degree of actuation in a more refined manner.

**FIGURE 10:**
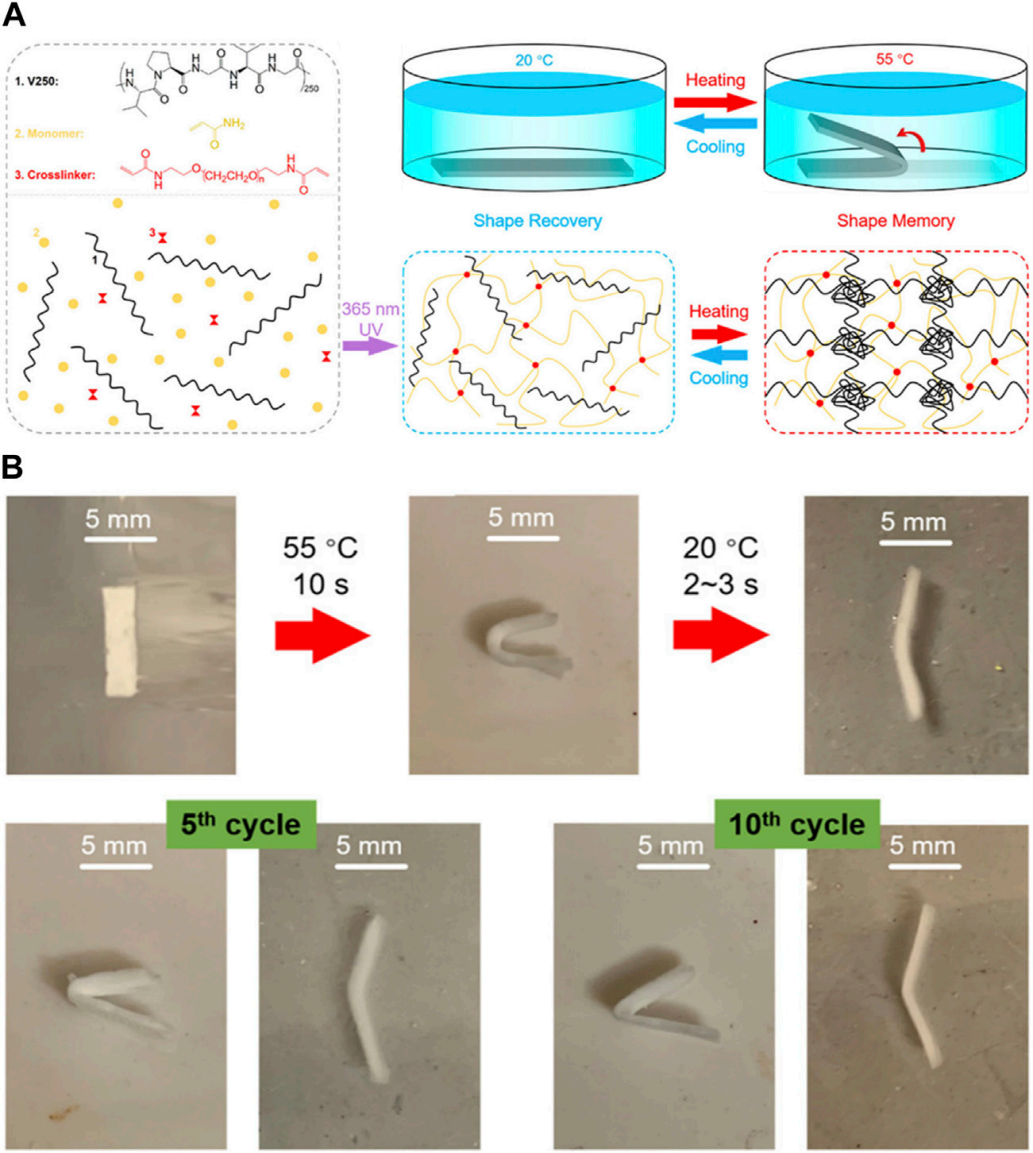
**(A)** Synthesis of semi-interpenetrating network hydrogel consisting of crosslinked polyacrylamide (PAAm) and ELP. ELP was physically incorporated into the PAAm network crosslinked by photoinitiated radical polymerization. Shape memory effect of the hydrogel could be imparted by programming the hydrogel at higher temperature via physical crosslinking between ELP molecules to hold the hydrogel in place. **(B)** Photographs of hydrogels demonstrating shape memory effect. The hydrogel could maintain the structure even after repeated shape deformations. Reproduced with permission from Zhang et al. (2020a). Copyright ^©^ 2020 American Chemical Society.

ELP has also been used to generate SMPs, in which the ability of ELP to undergo phase transition above LCST and crosslinking could serve as the soft segment. For example, Zhang et al. developed a semi-interpenetrating network hydrogel consisting of chemically crosslinked PAAm and physically-crosslinked ELP (Figure 10) Zhang et al. (2020b). The hydrogel was shown to display higher tensile strength at higher temperature, being able to maintain the structure without fracture and revert back to the original shape after extreme shape deformation. This indicated the strong physical crosslinking of ELP via hydrophobic interaction at higher temperature. In addition, the hydrogel could be programmed to a fixed shape without structural damage at 55°C above LCST, and the shape recovery took place at lower temperature. While synthetic polymers generally used for conventional SMPs have higher *T*_g_, necessitating significant thermomechanical energy for shape deformation, the transition temperature of ELP is much lower and the thermoresponsive deformation occurs naturally at higher temperature, requiring much less external energy for shape programming. It should be noted that it differs from the traditional SMPs in that the deformed hydrogel at higher temperature could not maintain its structure and reverted to its original shape at lower temperature.

**FIGURE 11:**
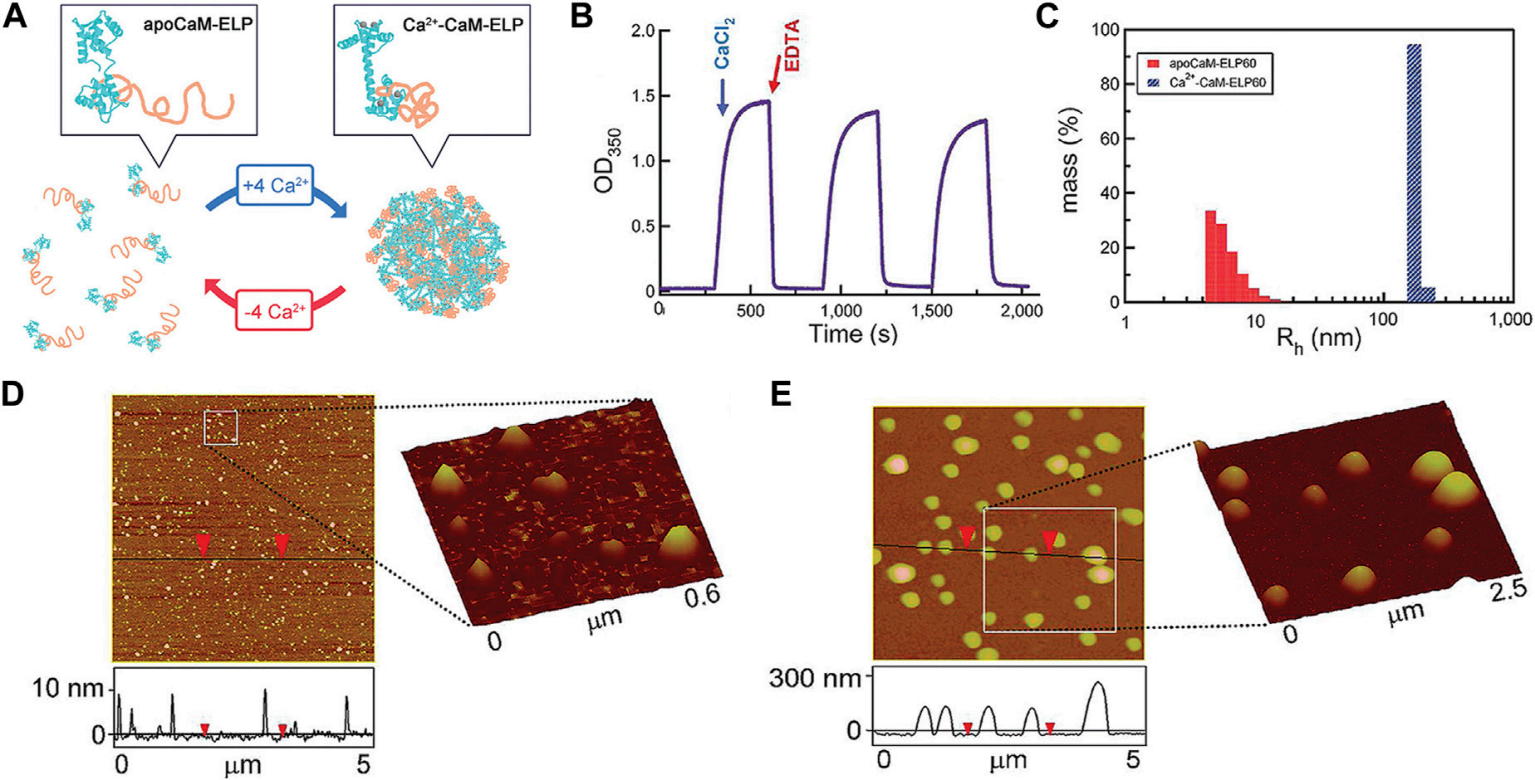
**(A)** Induction of self-assembly of apoCaM-ELP fusion protein to form large coacervates by binding with calcium (Ca^2+^-CaM-ELP). **(B)** The self-assembly by calcium and disassociation by removing calcium with chelation could be repeatedly performed. **(C)** The formation of self-assembled nanostructures was evaluated by the increase in the particle size (hydrodynamic radius). AFM images of **(D)** apoCaM-ELP and **(E)** Ca^2+^-CaM-ELP. Large coacervates were only detected for Ca^2+^-CaM-ELP. Reproduced with permission from Kim and Chilkoti, (2008). Copyright ^©^ 2008 American Chemical S.ociety.

The biocompatible nature of ELP, being a natural biopolymer, also allows the ELP-based actuators to be used for biomedical applications. For example, the thermoresponsive shape change of ELP could be used as a “switch” to induce conformational changes of nanostructures for drug delivery and biosensing applications. Kim et al. developed a fusion protein consisting of calmodulin (CaM) and ELP via recombinant DNA technology to fuse two genes (Figure 11) Kim and Chilkoti, (2008). CaM module served as the ligand binding domain, in which selective binding of calcium induced to the simultaneous phase transition of ELP module. Without calcium, the CaM-ELP undergoes phase transition above LCST to form self-assembled nanoparticles. With the addition of calcium, the binding of calcium with CaM module caused more extensive phase transition of the ELP module, leading to the formation of larger, micrometer-scale aggregates. This process was shown to be reversible by removing the calcium via chelation. Since many biological processes involve calcium ions, it could be envisioned that this fusion protein as a “control switch” to modulate the processes. Lee et al. synthesized a ABA-triblock copolymer consisting of ELP endblocks and CaM midblock, capable of thermoresponsive hydrogel formation Lee et al. (2022a). The mechanical properties and conformational changes were controlled by the binding of calcium to CaM midblock. The release of trifluoperazine as a co-ligand with calcium from the hydrogel showed significant cytotoxic effect on the cancer cells, demonstrating the potential as a cancer therapy.

Photocrosslinking is one of the most popular methods to create hydrogels. Like other biopolymers, ELP has been modified with photolabile groups to generate hydrogel via photocrosslinking. Compared to physical crosslinking of ELP copolymers, mechanical properties of photocrosslinked ELP hydrogels can be more efficiently and broadly tuned. Guo et al. developed ELP hydrogel by photocrosslinking methacrylic functionalized ELP (“ELP-MA”) Guo et al. (2022). The mechanical properties and thermoresponsive shape deformation could be controlled by the concentration and the degree of methacrylation of ELP. The hydrogel was demonstrated to be highly effective as a tissue adhesive and tissue engineering scaffolds.

## 4 Conclusion and perspective

Elastin has long fascinated biomedical researchers for its unique role in tissue physiology; controlling mechanical properties of ECM as well as providing biophysical cues to influence cellular behavior. Especially, elastin is largely responsible for the high mechanical strength and elasticity of biological tissue, which is originated from the coiled secondary structure of tropoelastin polypeptides that are enzymatically crosslinked. The tropoelastin consists of repeated pentapeptide units, rich in valine, glycine and proline that mediates hydrophobic interaction-driven coiled morphology above a certain transition temperature. Due to the difficulty of obtaining pure elastin from natural sources and the advantages of imparting desired functionalities, elastin-like polypeptide (ELP), which contains characteristic pentapeptide repeats and whose specific sequences can be precisely designed and produced by recombinant DNA technology, have been more widely utilized. The thermoresponsive phase transition behavior of ELP has proven to be quite useful for developing biomaterials, such as hydrogels, nanofibers and self-assembled nanostructures for biomedical applications including drug delivery and tissue engineering.

The ability of thermoresponsive polymeric materials to undergo shape deformation, such as widely popular poly(N-isopropylacrylamide) (PNIPAm), is being widely adopted to create actuators for soft robotics and multifunctional biomedical devices. Just as ELP has followed the footsteps of PNIPAm in the field of biomedical engineering, the recent research trend regarding ELP is suggesting that it is also being seriously considered as thermoresponsive soft actuators. The number of published reports is not significant at this point to imply that it is becoming a broad trend, but it is not surprising given that ELP itself has not reached the status of more popular biopolymers, such as alginate, chitosan, and gelatin for biomedical applications, largely due to the fundamental limitation of mass producibility. However, with its unique thermoresponsive properties and precise control of physicochemical properties that other biopolymers do not possess, ELP has found its niche in the field of biomedicine as a stimuli-responsive biomaterial. In the same manner, ELP has the potential to provide the similar attributes to developing soft actuators by providing new functionalities afforded by the genetic engineering.

Even though ELP can be produced in relatively large quantities via recombinant technology, it is still not economically viable enough to develop large-scale actuators solely using ELP. Furthermore, the mechanical strength of ELP is not high enough to develop robust structures required for actuators. Therefore, ELP is more ideally suited as a functional moiety to impart thermoresponsiveness and bioactivity to a known material. As the direction of the research in soft actuators is shifting towards developing multifunctional actuators with different modes of actuation and a diverse array of properties, it is also expected that more hybridization strategies would be utilized to generate ELP-based composite materials. As a burgeoning material system for soft actuators, the ELP-based actuators developed thus far have been mostly geared toward biomedical applications. However, with further maturation, it can be envisioned that the ELP-based soft actuators could also find their place in other areas, such as electronics and environmental engineering.
